# Supplementary material for: Ancient Egyptian mummy genomes suggest an increase of Sub-Saharan African ancestry in post-Roman periods
Source: Nat Commun. 2017 May 30;8:15694. doi: 10.1038/ncomms15694 (PMC5459999; doi:10.1038/ncomms15694)
Supplement: Supplementary Information — Supplementary Figures, Supplementary Tables, Supplementary Notes and Supplementary References [file ncomms15694-s6.pdf]

Supplementary Figures

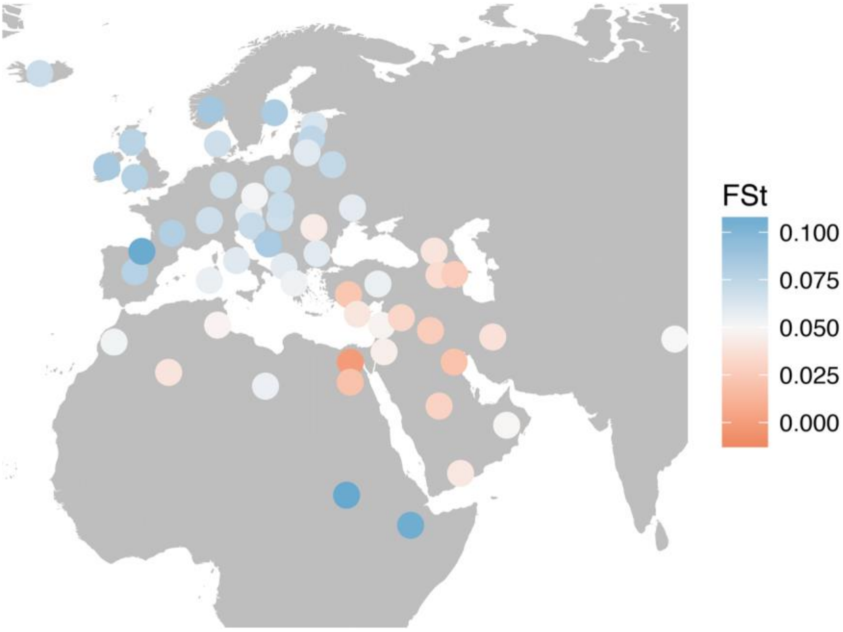

Supplementary Fig. 1: F_{ST} geographical mapping.
Analysis results of the HVR-1 modern populations in relation to our ancient meta-population of 90 ancient Egyptians. Blue values depict higher genetic distances, red values depict lower distances between the ancient meta-populations to present day populations in the respective area.

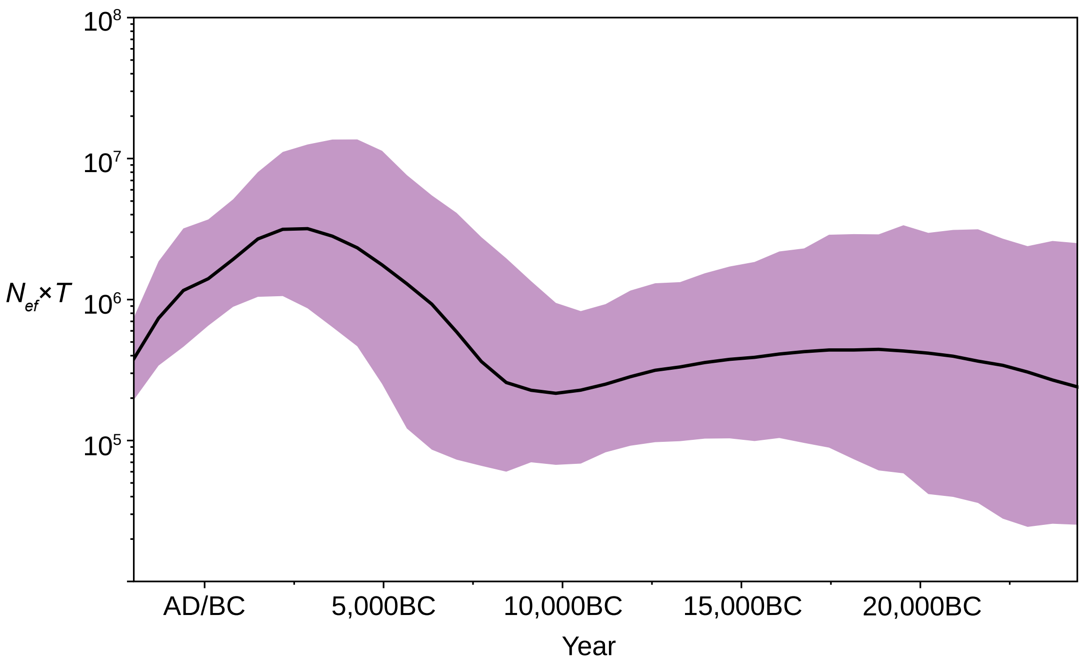

Supplementary Fig. 2: BEAST Effective Population Size Analysis.
Bayesian SkyGrid population size reconstruction based on 90 ancient and 135 modern Egyptian mitochondrial genomes. The thick black line indicates the estimated median female effective population size multiplied by generation time that need to be rescaled by 1:14.5 for the estimation of the studied population size (assuming 29 year generation time and equal male and female effective population sizes). Shaded violet area delineates the 95% Highest Posterior Density for the population size estimate. Estimates obtained using BEAST v 1.8.3. Plot generated with Tracer v 1.6.

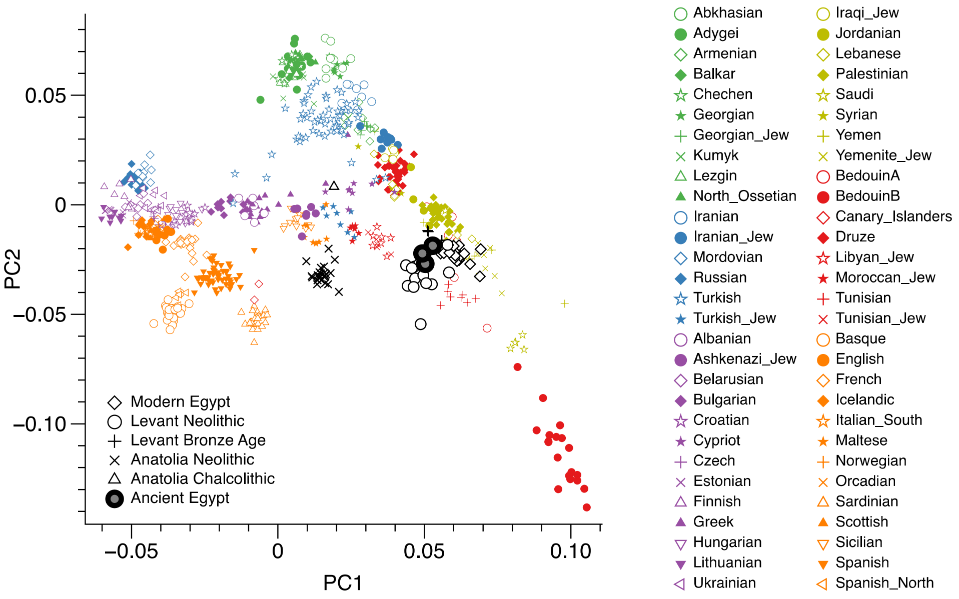

Supplementary Fig. 3: Principal component analysis using only European samples based on the nuclear genome-wide data obtained on three ancient samples.

The PCA was calculated as a projection to other published genomic data. A similar analysis as shown in Figure 4a with details is explained in Supplementary Note 2.

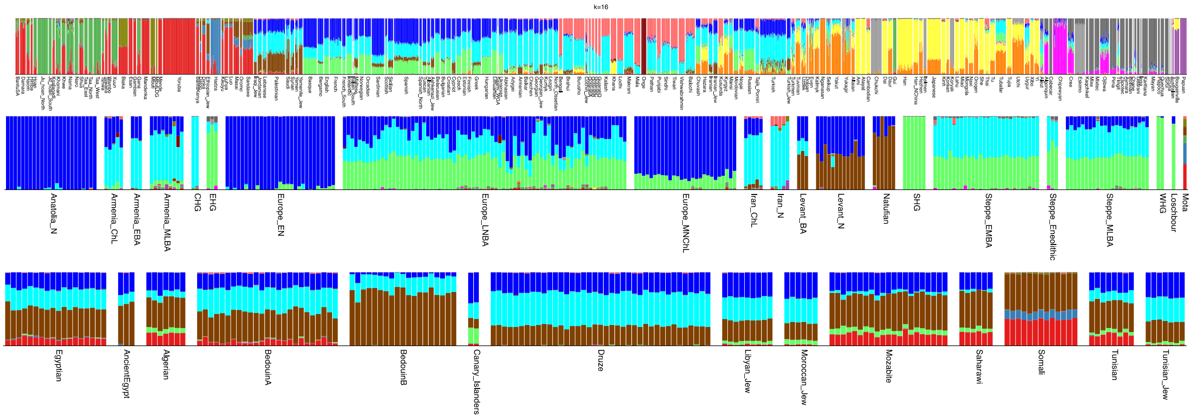

Supplementary Fig. 4: Complete results from the ADMIXTURE analysis using all samples in the merged data set.
Ancient samples are from Neolithic / Early-Neolithic (N, EN), Chalcolithic (ChL) or bronze age, early bronze age and middle bronze age (BA, EBA, MLBA) time.

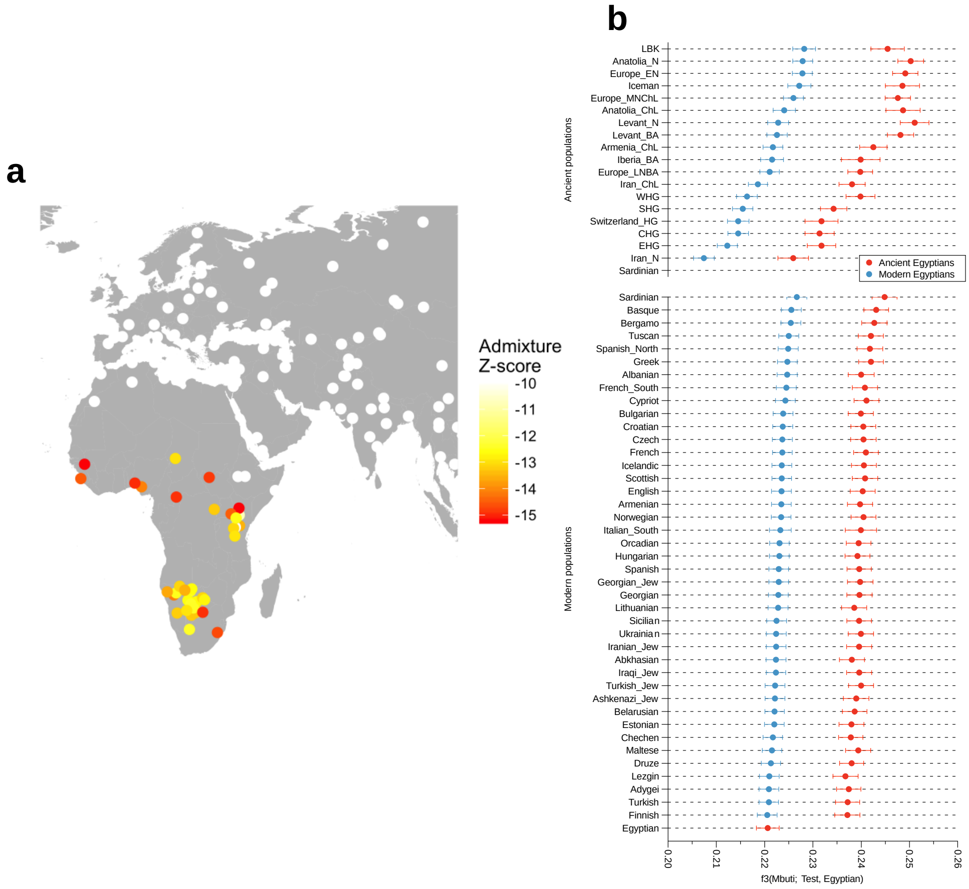

Supplementary Fig. 5: F3 statistics using only the uncontaminated sample JK2911.

A similar analysis as shown in Figure 5b/5c, but using only sample JK2911 as the Ancient Egyptian sample, instead of all ancient samples. Ancient samples are from Neolithic (N, EN), Chalcolithic (ChL) or Bronze Age (BA, EBA, MLBA) time periods as indicated in the suffix of the respective population names.

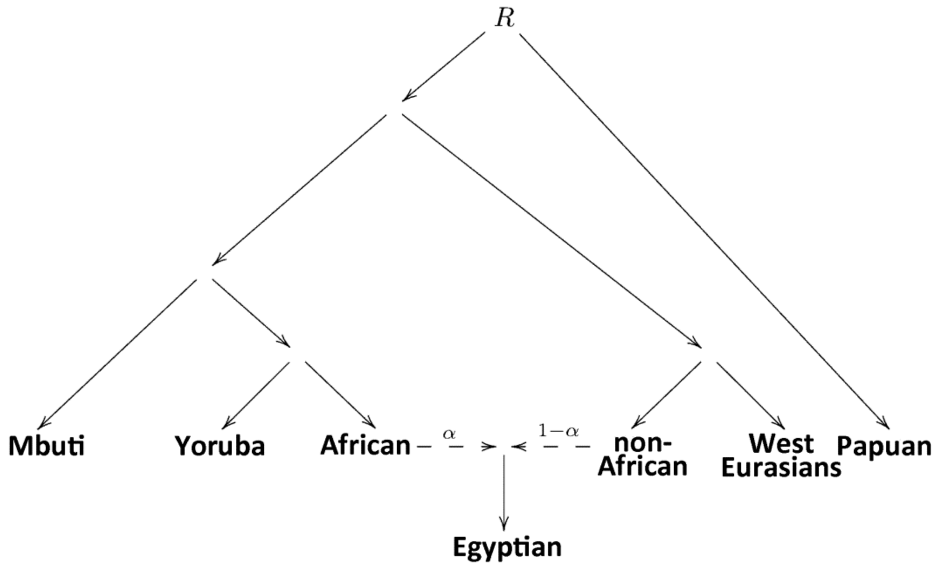

Supplementary Fig. 6: A phylogeny that we applied in f4-ratio estimation of African ancestry in Egyptians.

Supplementary Tables

Supplementary Table 1: Multiple sampled individuals.

Dark grey marked samples were included in the further analysis. Grey marked samples were not included in the analysis, due to one (better) sample from the same individual was used in the downstream analysis. However these fulfilled the required criteria as well. Samples with the lightest grey were not included in the further analysis due to missing the required criteria defined by us.

ID	Sample Name	Tissue type	avg mt Coverage	DMG 1st Base 3'	DMG 1st Base 5'	final cont est	low	high	C14 Date	Time Period
**1543**	1543BM	bone	195.49	0.09	0.09	0.01	0	0.02	54 - 124 AD	Roman Period
**1543**	JK2139	soft tissue	438.70	0.10	0.11	0.01	0.00	0.02	54 - 124 AD	Roman Period
**1554**	1554BM	bone	748.10	0.08	0.08	0.13	0.12	0.14	402 - 385 BC	Pre-Ptolemaic Period
**1554**	JK2140	soft tissue	3.40	0.15	0.13	NA	NA	NA	402 - 385 BC	Pre-Ptolemaic Period
**1564**	1564BM	bone	328.54	0.07	0.07	0.01	0.00	0.02	358 - 204 BC	Ptolemaic Period
**1564**	1564TM	tooth	211.32	0.11	0.11	0.01	0.01	0.02	358 - 204 BC	Ptolemaic Period
**1564**	JK2141	soft tissue	117.53	0.22	0.22	0.01	0.00	0.02	358 - 204 BC	Ptolemaic Period
**1585**	1585BM	bone	170.61	0.12	0.12	0.01	0.01	0.02	382 - 234 BC	Ptolemaic Period
**1585**	JK2142	soft tissue	10.97	0.24	0.26	0.01	0.00	0.02	382 - 234 BC	Ptolemaic Period
**1608**	1608BM	bone	2597.81	NF	NF	0.04	0.05	0.05	801 - 777 BC	Pre-Ptolemaic Period
**1608**	JK2143	soft tissue	32.72	0.20	0.20	0.01	0.00	0.02	801 - 777 BC	Pre-Ptolemaic Period
**1622**	1622BM	bone	35.72	0.15	0.15	0.01	0.01	0.02	806 - 784 BC	Pre-Ptolemaic Period
**1622**	JK2144	soft tissue	5.49	0.23	0.23	NA	NA	NA	806 - 784 BC	Pre-Ptolemaic Period
**1614**	JK2917	tooth	25.92	0.15	0.17	0.01	0.00	0.02	753 - 544 BC	Pre-Ptolemaic Period
**1614**	JK2923	bone	56.41	0.15	0.16	0.01	0.00	0.02	753 - 544 BC	Pre-Ptolemaic Period
**1573**	JK2868	bone	17.42	0.12	0.08	0.48	0.47	0.49	97 - 2 BC	Ptolemaic Period
**1573**	JK2888	tooth	567.83	0.08	0.09	0.01	0.00	0.02	97 - 2 BC	Ptolemaic Period
**1648**	JK2969	tooth	14.22	0.01	0.02	NA	NA	NA	87 BC - 1 AD	Ptolemaic Period
**1648**	JK2960	bone	13.39	0.25	0.26	0.01	0.00	0.02	87 BC - 1 AD	Ptolemaic Period
**1627**	JK2982	tooth	394.03	0.18	0.18	0	0	0.005	cal BC 92-1	Ptolemaic Period
**1627**	JK2983	bone	93.42	0.10	0.09	0	0	0.025	cal BC 92-1	Ptolemaic Period

Supplementary Table 2: Results of the genetic distance computation with Arlequin between our three investigated populations from ancient Egypt and two modern populations with available mitochondrial genomes from Egypt and Ethiopia.

Differences between the three individual populations are fairly small, therefore we grouped all three ancient populations together. Significant p-Values (<= 0.05) are highlighted in green. Pairwise Fst (top) and significance values (bottom) were computed based on the mitochondrial DNA. The corresponding p-values (upper diagonal, bottom matrix) were corrected (lower diagonal, in italics, bottom matrix) using the Benjamini-Hochberg method as provided in the p.adjust function using GNU R.

	N
Pre-Ptolemaic	44
Ptolemaic	27
Roman	19
Egypt Modern Pagani	100
Egypt Modern Kujánova	35
Ethiopia Modern	120
Total	**345**

Distance Method	Tamura & Nei
Gamma Shape:	0,98900

Individual Populations

**FSt**	Ancients			Modern Egypt		
	Pre-Ptolemaic	Ptolemaic	Roman	EGY Pagani	EGY Kujanova	ETH Pagani
Pre-Ptolemaic	0.00000					
Ptolemaic	0.00155	0.00000				
Roman	-0.00725	-0.00078	0.00000			
EGY Pagani	0.01149	0.00309	0.00081	0.00000		
EGY Kujanova	0.04385	0.03350	0.04026	0.02526	0.00000	
ETH Pagani	0.09654	0.08281	0.06996	0.06016	0.05365	0.00000

**p Values**	Ancients			Modern Egypt		
	Pre-Ptolemaic	Ptolemaic	Roman	EGY Pagani	EGY Kujanova	ETH Pagani
Pre-Ptolemaic	*	0.34277+-0.0174	0.6695+-0.0060	0.02148+-0.0060	0.00098+-0.0010	0.00000+-0.0000
Ptolemaic	0.41916	*	0.43359+-0.0146	0.22949+-0.0147	0.02051+-0.0047	0.00000+-0.0000
Roman	0.6695	0.46456	*	0.36328+-0.0119	0.03711+-0.0062	0.00000+-0.0000
EGY Pagani	0.0358	0.31294	0.4191692	*	0.00293+-0.0016	0.00000+-0.0000
EGY Kujanova	0.00245	0.0358	0.055665	0.00627857	*	0.00000+-0.0000
ETH Pagani	0	0	0	0	0	*

Grouped Ancient Populations

**FSt**	Ancients	Egypt Modern	Ethiopia Modern
Ancients	*		
Egypt Modern	0.01363	*	
Ethiopia Modern	0.10257	0.0565	*

**p Values**	Ancients	Egypt Modern	Ethiopia Modern
Ancients	*	0.0000+-0.0000	0.0000+-0.0000
Egypt Modern	*0.00000*	*	0.0000+-0.0000
Ethiopia Modern	*0.00000*	*0.00000*	*

**FSt**	Ancients	Modern Egypt		
		EGY Pagani	EGY Kujanova	ETH Pagani
Ancients	*			
EGY Pagani	0.01148	*		
EGY Kujanova	0.0516	0.02526	*	
ETH Pagani	0.10257	0.06016	0.05365	*

**p Values**	Ancients	Modern Egypt		
		EGY Pagani	EGY Kujanova	ETH Pagani
Ancients	*	0.00293+-0.0016	0.00000+-0.0000	0.00000+-0.0000
EGY Pagani	0.00293	*	0.00195+-0.0014	0.00000+-0.0000
EGY Kujanova	0	0.00234	*	0.00000+-0.0000
ETH Pagani	0	0	0	*

Supplementary Table 3: Y-Chromosomal haplotype results.

Sample ID	*Y-Haplogroup*	*Comment*
JK2134	J	This individual was assigned to haplogroup J based on mutations: CTS8938/PF4577: 18567169T→G, F2817/PF4579: 18695159C→T, F4299/PF4589: 21144431T→A, S22619/Z7820: 21144432C→A, F4300: 21144433T→A, YSC0000228: 22172960G→T, L778/PF4616/YSC0000236: 23088142T→C.
JK2888	E1b1b1a1b2	This individual was assigned to haplogroup E1b1b1a1b2 based on mutation: V22: 6859957T→C, and to upstream E1b1b1a: CTS2661: 14410669C→T and E1b1b1: PF1619: 13848122T→C, CTS2620: 14393170A→C, M5360: 23618826C→T.
JK2911	J	This individual was assigned to haplogroup J based on mutations: CTS687/PF4503:6953311A→T, CTS1250/PF4510/YSC0001255:7296343G→T, PF4513/NA:7759610C→T, PF4519/NA:8669451C→G, PF4524/NA:10009851G→A, PF4530/NA:13597365C→T, CTS2769/PF4538:14476551T→A, F1973/PF4546/YSC0001304:15581303G→A, F2114/PF4551:16262942G→A, CTS5628/PF4555:16401405C→G, CTS5678/PF4556:16427564A→T, F2502/PF4564:17495914G→A, CTS7738/PF4568:17637446T→C, CTS7832/PF4569:17693210A→G, F2769/PF4576:18552360G→C, F2973/PF4585/YSC0001312:19194316C→T, F4299/PF4589:21144431T→A, S22619/Z7820:21144432C→A, F4300/NA:21144433T→A, F3176/PF4592/YSC0001314:21329083T→C, PF4595/NA:21858778C→A, YSC0000228/NA:22172960G→T, M304/Page16/PF4609:22749853A→C, L778/PF4616/YSC0000236:23088142T→C, CTS11571/PF4617:23163701C→A, CTS11750/PF4618/YSC0001250:23250894C→T, CTS12047/YSC0001253:23443976A→G

Supplementary Table 4: Effective population size estimates calculated with BEAST.

Size estimates from the Bayesian SkyGrid analysis for the population of Abusir El-Meleq. The values were calculated assuming 29 year generation time and an equal male to female ratio in the population. Time points are population size inflection points inferred during the analysis.

Estimation time point	Cultural period	Population size estimate			
		Mean	Median	Lower 95% HPD	Upper 95% HPD
612 AD	Roman	83.769	79.985	31.864	220.377
82 BC	Ptolemaic	100.288	96.999	44.991	255.025
776 BC, 1469 BC (averaged estimates)	Pre-Ptolemaic	165.505	159.261	66.663	453.713
	**average**	**116.521**	**112.082**	**47.839**	**309.705**

Supplementary Table 5: D-Statistics.

We computed D statistics of the form D(Modern Egyptian, Ancient Egyptian; X, Mbuti) to find populations that the Ancient Egyptians share more ancestry with compared to modern Egyptians.

Population	D statistics	Z-score
Levant_BA	0.0507	15.175
Levant_N	0.0543	14.924
Anatolia_N	0.0437	14.714
Europe_EN	**0.0425**	14.691
Europe_MNChL	0.0421	14.648
Europe_LNBA	0.03630	13.387
Armenia_ChL	0.04130	12.671
Egyptian_Pag	0.0312	12.621
Steppe_MLBA	0.0355	12.357
BedouinB	0.0322	12.256
Palestinian	0.031	12.121
Sardinian	0.0337	12.042
Yemenite_Jew	**0.034**	11.971
Iran_ChL	0.0382	11.927
Greek	0.03210	11.844
Lebanese	0.03190	11.842
Druze	0.0309	11.812
BedouinA	0.0295	11.81
Basque	0.0325	11.749
Turkish	0.0304	11.701

Supplementary Table 6: f4-ratio based estimates of African ancestry (α) in Egyptians.

The "std.err" is the standard error estimated using jackknifes. "Z" is the Z-score for the estimation.

Admixed populations	West Eurasian sources	African ancestry (α)	std.err	Z
Egyptian	French	0.17165	0.004346	39.496
AncientEgyptians	French	0.092278	0.010924	8.447
Egyptian	Anatolia_N	0.142236	0.004902	29.016
AncientEgyptians	Anatolia_N	0.06112	0.011791	5.184
Egyptian	WHG	0.208021	0.00873	23.827
AncientEgyptians	WHG	0.12929	0.013958	9.263
Egyptian	EHG	0.209352	0.009337	22.421
AncientEgyptians	EHG	0.138128	0.01385	9.973
Egyptian	SHG	0.205056	0.007641	26.835
AncientEgyptians	SHG	0.134262	0.013155	10.206
Egyptian	Iran_N	0.138289	0.010708	12.914
AncientEgyptians	Iran_N	0.056609	0.016074	3.522
Egyptian	CHG	0.149992	0.00995	15.074
AncientEgyptians	CHG	0.073004	0.01561	4.677
Egyptian	MA1	0.22376	0.011275	19.846
AncientEgyptians	MA1	0.148986	0.017456	8.535

Supplementary Table 7: The African admixture proportions estimated using qpAdm.
Here “p” refers to the P-value for rank=1 and “std.err” is the standard error estimated using jackknifes.

Admixed populations	West Eurasian sources	African ancestry (α)	std.err	p
Egyptian	French	0.161	0.004	0.096
AncientEgyptians	French	0.079	0.013	0.449
Egyptian	Anatolia_N	0.13	0.005	0
AncientEgyptians	Anatolia_N	0.041	0.014	0.48
Egyptian	WHG	0.174	0.01	0.735
AncientEgyptians	WHG	0.096	0.017	0.53
Egyptian	EHG	0.177	0.011	0.504
AncientEgyptians	EHG	0.089	0.018	0.519
Egyptian	SHG	0.178	0.008	0.721
AncientEgyptians	SHG	0.104	0.016	0.795
Egyptian	Iran_N	0.109	0.012	0.821
AncientEgyptians	Iran_N	0.029	0.02	0.957
Egyptian	CHG	0.144	0.01	0.436
AncientEgyptians	CHG	0.066	0.017	0.65
Egyptian	MA1	0.172	0.014	0.838
AncientEgyptians	MA1	0.09	0.022	0.903

Supplementary Table 8: Admixture time and lower bound of proportion estimated by ALDER.

Population	N	2-ref decay for Yoruba and French (generations)	2-ref	1-ref decay for Yoruba (generations)	1-ref	Mixture fraction % lower bound

			Z-score		Z-score	
Egyptian	18	24.61±5.90	4.17	23.42±3.86	6.06	6.8±0.7

Supplementary Table 9: Allele information of SNPs thought to be affected by selection in samples.

Only high-quality (q>30) bases are counted. rs4988235 is responsible for lactase persistence in Europe. The SNPs at *SLC24A5* and *SLC45A2* are responsible for light skin pigmentation. The SNP at *EDAR* affects tooth morphology and hair thickness. The SNP at *HERC2* is the primary determinant of light eye color in present-day Europeans.

		LCT	SLC45A2	SLC24A5	EDAR	HERC2
	SNP	rs4988235	rs16891982	rs1426654	rs3827760	rs12913832
	Ancestral	G	C	G	A	A
	Derived	A	G	A	G	G
JK2134	Coverage					
	Derived allele frequency					
JK2888	Coverage			1	1	
	Derived allele frequency			100%	0%	
JK2911	Coverage	10	7	2	3	2
	Derived allele frequency	0%	0%	100%	0%	0%

Supplementary Notes

Note 1: *f4*-ratio based estimation of African ancestry in present-day and ancient Egyptians

Chuanchao Wang

The African admixture proportions in present-day and ancient Egyptians are estimated in model-based clustering by ADMIXTURE in successive models with increasing number of "ancestral populations" (increasing K). In this section, we confirmed these estimates using a simpler f4-ratio approach (*39*) applied to the proposed graph below (Supplementary Fig. 6).

We used different West Eurasian populations to act as the proxies for the non-African sources. We observed an increase of Yoruba related African ancestry in present-day Egyptians compared with our ancient samples. The present-day Egyptians are suggested to have about 14% to 21% African ancestry, while the proportions in ancient Egyptians are only 6% to 15%, as also depicted in Supplementary Table 6.

<u>Admixture proportion estimation using qpAdm</u>

In this section, we used qpAdm (*35, 71*) to estimate the African admixture proportions in ancient and present-day Egyptians. qpAdm estimates ancestry proportions from two or more proxy source populations assuming the proxies are more closely related to the true source populations than a set of proposed outgroups. It also provides a P-value for the consistency of this hypothesis with the data.

We use the following set of outgroups including the extinct Denisovan, Upper Paleolithic Eurasian (Ust_Ishim), and three eastern non-African population (Onge, Papuan, and Australian). We used Yoruba and different West Eurasian groups as proxies for the source populations. The results are consistent with ADMIXTURE and f4-ratio that present-day Egyptians share more ancestry with

Africans compared with ancient Egyptians, as shown in detail in Supplementary Table 7.

Dating gene flow from Africans into present-day Egyptians

In this section, we estimated admixture time and lower bounds on the admixture proportion for the observed African ancestry using the linkage disequilibrium (LD)-based admixture inference implemented in ALDER (*40*). We computed weighted LD curves with present-day Egyptians as the test populations and Yoruba and French as references. As French might not be a close surrogate for all diverse populations in West Eurasia that might contribute to Egyptians, we also took advantage of the one-reference inference capabilities of ALDER to only use Yoruba as reference.

The average admixture time for present-day Egyptians is 24 generations ago (about 700 years ago assuming 29 years a generation (*72*)), as further illustrated in Supplementary Table 8. We estimated mixture fractions of at least 6.8% Yoruba-related ancestry for those present-day Egyptians. Changing the starting point of the LD fit does not qualitatively affect the results.

We caution that the date estimates might not reflect the initial African admixture in present-day Egyptians; instead, it is an average date of population mixture. If the admixture did not happen immediately when two populations met, or occurred many times over an extended period, the true start of mixture would be more ancient.

Note 2: Nuclear data capture analysis

Stephan Schiffels, Alexander Peltzer, Anja Furtwängler, Verena J. Schuenemann

Sample selection for nuclear capture

For the nuclear capture, we chose 40 samples from our mitochondrial results to prepare libraries for further nuclear capture. Our choice was based on obtained

coverage of the respective sample, low mitochondrial contamination (<3%) and significant damage patterns to only include samples with patterns of ancient origin into our nuclear capture. Furthermore, we estimated potential yield based on endogenous content in our previously screened samples. The final choice of these samples can be seen in Supplementary Data 2.

Capture

Samples that were further investigated for nuclear DNA content underwent Uracil-DNA-Glycosylase (UDG) treatment. Two aliquots of 20 µl of each DNA extract of the samples selected for the enrichment of nuclear DNA were used to generate two sequencing libraries per sample after Meyer and Kircher (*22*). To reduce the effect of damage-derived C to T and G to A misincorporations typical for ancient DNA, the extracts were treated with USER™ enzyme (New England Biolabs) containing Uracil-DNA-Glycosylase and Endonuclease VIII (*27*).

To each library a library specific set of indexing sequences were added to each end of the library fragments by amplification with tailed primers in a 10 cycle PCR (*23*).

For all indexed libraries an additional amplification was performed. For each library 20 µl were used for four reactions with a total volume of 100 µl respectively and the following concentrations: 1x AccuPrime™ Pfx Reaction Mix, 0,3 µm IS 5, 0.3 µm IS 6 and 0.02 Units/µl AccuPrime™ Pfx DNA Polymerase (Invitrogen). The amplification product was purified with the MinElute Purification Kit (Qiagen) after the manufactures instruction and an aliquot was quantified with the Agilent 2100 Bioanalyzer according to the manufactures instructions using the Agilent 2100 Bioanalyzer DNA1000 chip (Agilent Technologies) to determine the distribution of the fragment size and the concentration of the library.

The non-UDG and UDG treated libraries were enriched by hybridization to probes targeting approximately 1.24 million genomic SNPs. The target SNPs consist of panel 1 and 2 as described in Mathieson et al. (*41*) and Fu et al. (*12*), a large proportion of which are also present on the Affymetrix Human Origins, the Illumina 610-Quad and the Affymetrix 50k array. The probes had a length of 52nt covering a region of 105nt flanking the target SNPs in the center. The

enrichment was performed as described in Fu et al. (*25*). The two UDG treated libraries per sample were pooled for the capture while the non-UDG treated libraries were captured separately. After the last purification all enriched libraries were pooled for sequencing.

PCA

We performed principal component analysis on the joined data set using the "smartpca" software from the Eigensoft package (*63*). For the plot shown in Supplementary Fig. 3, we used a selected set of European populations: Abkhasian, Adygei, Albanian, Armenian, Balkar, Basque, BedouinA, BedouinB, Belarusian, Bulgarian, Canary_Islanders, Chechen, Croatian, Cypriot, Czech, Druze, English, Estonian, Finnish, French, Georgian, Greek, Hungarian, Icelandic, Iranian, Italian_South, Ashkenazi_Jew, Georgian_Jew, Iranian_Jew, Iraqi_Jew, Libyan_Jew, Moroccan_Jew, Tunisian_Jew, Turkish_Jew, Yemenite_Jew, Jordanian, Kumyk, Lebanese, Lezgin, Lithuanian, Maltese, Mordovian, North_Ossetian, Norwegian, Orcadian, Palestinian, Russian, Sardinian, Saudi, Scottish, Sicilian, Spanish_North, Spanish, Syrian, Turkish, Ukrainian. For the plot shown in Figure 4a, we added the following African populations: Ethiopian_Jew, Dinka, Luhya, Algerian, Mozabite, Saharawi, Somali, Yoruba, Mota, Mandenka, Biaka.

Note 3: Mitochondrial DNA sequence processing and alignment

Alexander Peltzer, Kay Nieselt, Wolfgang Haak

Adapter sequences were trimmed from the 3' ends of reads using Clip&Merge v1.7 (*57*), requiring an overlap of 10 bp between the adapter and the corresponding read for subsequent read merging. A minimum read length filtering of 25 nt has been used to ensure that reads, which were too short, were not incorporated into the further analysis. The resulting sequences have been aligned using the Burrows-Wheeler-Aligner (BWA) version 0.7.12 (*73*), with the parameters –n 0.01 and –l 1000 to disable seeding and allow more mismatches due to the age of the samples. All reads have been aligned against the GRCh37

build of the human genome with the Reconstructed Sapiens Reference Sequence (RSRS) (*74*) to account for NUMTs.

Note 4: Sequence based mitochondrial analysis

Alexander Peltzer, Martyna Molak, Wolfgang Haak

<u>Modern comparative data</u>

To allow for further comparisons between the ancient samples from Pre-Ptolemaic, Ptolemaic and Roman time periods in Egypt, we used an extensive private database of 37,368 HVR-I sequences (nucleotide positions 16,059-16,400) from contemporary populations in Europe, the Near East and Africa (*29*). These have been pooled according to geographical information provided in the original publications and collected from literature. All sequence data used were updated from the original publication to the mtDNA phylogeny of phylotree.com v.17 (*75*).

Our reference dataset consisted of these populations:

I. Populations from central, eastern, western and southern Europe, representing the genetic context of Slovenia, Hungary, Italy, France, Serbia, England, Faroe-Islands, Finland, Norway, Sweden, Spain and Iceland.
II. Populations from North-Africa, representing the genetic context of Egypt, Mauretania, Tunisia, West-Sahara and Morocco.
III. Populations from Sub-Saharan Africa, represented by populations from Sudan, Ethiopia, Burkina-Faso, Cameroon and Guinea.
IV. Populations from the Near East, represented by populations from Syria, Iran, Iraq, Turkey, Yemen, Kuwait, United Arab Emirates, Lebanon, Israel, Oman, Saudi-Arabia, Qatar and Jordan.
V. Populations from the Caucasus and West Asia, represented by populations from Georgia, Armenia and Pakistan.

Citations for the studies reporting the original HVR-1 sequences are listed in Supplementary Data 4.

Geographical mapping

Similar to our approach in the MDS analysis, we evaluated the geographic distribution of mtDNA variance using a geographical mapping of our calculated F_{ST} values on our HVR-1 dataset and our corresponding ancient populations. Genetic distances were computed in Arlequin v.3.5.2.2 and afterwards combined with longitudes and latitudes and then subsequently mapped to a geographic map. We applied the R packages *ggmap* and *ggplot2* in a custom script to plot F_{ST} values to our geographic setting (*76*), creating a map of F_{ST} shown in Supplementary Fig. 1. For each of the populations used in this study, we defined a point of reference, which described best the available geographic information. In cases where we did not have exact geographic information, we used the innermost longitudinal / latitudinal value defined for the respective country.

Note 5: Frequency based mitochondrial analysis

Alexander Peltzer, Wolfgang Haak, Kay Nieselt

Test of population continuity

We followed an approach first used and defined by Brandt et al. (*29*) by first generating counts of 22 haplogroups determined manually to be most descriptive for our three ancient populations. Our priors on c (see Supplementary Data 5) were set based on the number of generations between two selected populations to test, with an assumed generation length of 25 years for our ancient populations. To determine potential influences of chosen effective population size N_e on our computations, we iteratively evaluated a range of population sizes for the specific region (see Supplementary Data 5) and investigated whether these changed our analysis significantly. The basic assumption of the method described by Brandt et al. (*29*) is to test a model of population continuity, e.g. compare allele frequencies of our ancient population with modern typed data from the same location. As Brandt et al. (*29*) already state, the idea is based on the assumption that mtDNA can only obtain new alleles by migration from an outside source and not by genetic drift only. The idea is therefore to test whether a model of genetic drift fits to our given dataset

and then test whether this model fits the data or has to be rejected. Internally this is done using a hypergeometric distribution, which is then evaluated using a MCMC package implemented in the scope of Brandt et al (*29*). We used the dataset of 120 Ethiopian and 100 Egyptian modern mtDNA genomes from Pagani et al. (*17*) to determine whether we can detect genetic discontinuity between ancient and modern Egypt. However, the method was only able to detect strong signs of discontinuity between our ancient populations and modern Ethiopians. For modern Egyptians, neither a significant value supporting discontinuity nor continuity was observed (see Supplementary Data 5). To ensure that the prior mean on the drift parameter t/N (where t is the number of generations separating the populations and N is the effective population size) did not influence our results, we chose our N based on historic written data (see Supplementary Data 5) and furthermore evaluated a range of N=(20K, 50K, 70K, 100K) effective population to account for possible wrong choices on N in our basic assumptions.

Note 6: Y-chromosomal & phenotypic analysis
Chuanchao Wang, Alexander Peltzer

The current distribution of E1b1b1 in North Africa could also be caused by the back migration from the Near East to Africa that have already been proposed by several authors (*77-79*). The high frequencies of haplogroup R1-M173 in Cameroon also supported the back migration from Eurasia to Africa (*80*). Since it's still unclear whether E1b1b evolved in Northeast Africa or the Near East, we were deciding against attempting to conclude whether the two haplogroups provide information about different paternal origin information in our three Mummy samples.
For our phenotypic analysis, we investigated a set of SNPs thought to be affected by selection in our samples. Only high-quality ($q > 30$) bases were counted. We were able to find derived alleles for the genes SLC24A5 (rs1426654), which are known to be responsible for lighter skin pigmentation in JK2888 and JK2911. Our further tests whether the genes SLC45A2 (rs16891982), LCT (rs4988235), EDAR (rs3827760) and HERC2 (rs12913832) revealed no derived alleles for

both JK2888 and JK2911. For JK2134, no sufficient coverage after quality filtering was given at the specific sites, which is why the analysis revealed no further clues (see Supplementary Table 6 for details). LCT is responsible for lactase persistence in Europe (*81, 82*). The SNPs at SLC24A5 and SLC45A2 are responsible for lighter skin pigmentation (*83*). The SNP at EDAR affects tooth morphology and hair thickness (*84, 85*). The SNP at HERC2 is the primary determinant of light eye colour in present-day Europeans (*86, 87*).

Supplementary References

1. I. Shaw, The Oxford History of Ancient Egypt. *Oxford University Press*, (2000).
2. C. Riggs, The Beatiful Burial in Roman Egypt: Art, Identity, and Funerary Religion. *Oxford University Press*, (2005).
3. S. Coussement, Because I Am Greek: Polyonymy as an Expression of Ethnicity in Ptolemaic Egypt. *Studia Hellenistica 55, Peeters Publishers, Leuven*, (2016).
4. Y. Broux, Double Names and Elite Strategy in Roman Egypt. *Studia Hellenistica 54, Peeters Publishers, Leuven*, (2016).
5. D. Shriner, S. O. Keita, Migration Route Out of Africa Unresolved by 225 Egyptian and Ethiopian Whole Genome Sequences. *Frontiers in genetics* **7**, 98 (2016).
6. S. Paabo, Molecular cloning of Ancient Egyptian mummy DNA. *Nature* **314**, 644-645 (1985).
7. S. Paabo, Ancient DNA: extraction, characterization, molecular cloning, and enzymatic amplification. *Proceedings of the National Academy of Sciences of the United States of America* **86**, 1939-1943 (1989).
8. M. T. Gilbert *et al.*, Long-term survival of ancient DNA in Egypt: response to Zink and Nerlich (2003). *Am J Phys Anthropol* **128**, 110-114; discussion 115-118 (2005).
9. I. Marota, C. Basile, M. Ubaldi, F. Rollo, DNA decay rate in papyri and human remains from Egyptian archaeological sites. *Am J Phys Anthropol* **117**, 310-318 (2002).
10. Z. Hawass *et al.*, Ancestry and pathology in King Tutankhamun's family. *Jama* **303**, 638-647 (2010).
11. E. D. Lorenzen, E. Willerslev, King Tutankhamun's family and demise. *Jama* **303**, 2471; author reply 2473-2475 (2010).
12. R. Khairat *et al.*, First insights into the metagenome of Egyptian mummies using next-generation sequencing. *Journal of applied genetics* **54**, 309-325 (2013).
13. A. W. Briggs *et al.*, Patterns of damage in genomic DNA sequences from a Neandertal. *Proceedings of the National Academy of Sciences of the United States of America* **104**, 14616-14621 (2007).

14. M. Stoneking, J. Krause, Learning about human population history from ancient and modern genomes. *Nature reviews. Genetics* **12**, 603-614 (2011).
15. G. Renaud, V. Slon, A. T. Duggan, J. Kelso, Schmutzi: estimation of contamination and endogenous mitochondrial consensus calling for ancient DNA. *Genome Biol* **16**, 224 (2015).
16. B. M. Henn *et al.*, Genomic ancestry of North Africans supports back-to-Africa migrations. *PLoS genetics* **8**, e1002397 (2012).
17. L. Pagani *et al.*, Tracing the route of modern humans out of Africa by using 225 human genome sequences from Ethiopians and Egyptians. *Am J Hum Genet* **96**, 986-991 (2015).
18. A. Lalremruata *et al.*, Molecular identification of falciparum malaria and human tuberculosis co-infections in mummies from the Fayum depression (Lower Egypt). *PloS one* **8**, e60307 (2013).
19. B. Welte, Zeitzeugen aus dem Wüstensand- die altägyptischen Mumienschädel aus Abusir el-Meleg *Dissertation, BioArchaeologica no. 6, Marie-Leidorf Verlag*, (2016).
20. F. Dunand, R. Lichtenberg, Mummies and Death in Egypt. *New York: Cornell University Press*, (2006).
21. T. M. Nicholson *et al.*, Enlightening the past: analytical proof for the use of Pistacia exudates in ancient Egyptian embalming resins. *Journal of separation science* **34**, 3364-3371 (2011).
22. M. Meyer, M. Kircher, Illumina sequencing library preparation for highly multiplexed target capture and sequencing. *Cold Spring Harbor protocols* **2010**, pdb.prot5448 (2010).
23. M. Kircher, S. Sawyer, M. Meyer, Double indexing overcomes inaccuracies in multiplex sequencing on the Illumina platform. *Nucleic Acids Res* **40**, e3 (2012).
24. T. Maricic, M. Whitten, S. Paabo, Multiplexed DNA sequence capture of mitochondrial genomes using PCR products. *PloS one* **5**, e14004 (2010).
25. Q. Fu *et al.*, DNA analysis of an early modern human from Tianyuan Cave, China. *Proceedings of the National Academy of Sciences of the United States of America* **110**, 2223-2227 (2013).
26. Q. Fu *et al.*, An early modern human from Romania with a recent Neanderthal ancestor. *Nature* **524**, 216-219 (2015).
27. A. W. Briggs *et al.*, Removal of deaminated cytosines and detection of in vivo methylation in ancient DNA. *Nucleic Acids Res* **38**, e87 (2010).
28. L. Excoffier, H. E. Lischer, Arlequin suite ver 3.5: a new series of programs to perform population genetics analyses under Linux and Windows. *Mol Ecol Resour* **10**, 564-567 (2010).
29. G. Brandt *et al.*, Ancient DNA reveals key stages in the formation of central European mitochondrial genetic diversity. *Science* **342**, 257-261 (2013).
30. M. Kujanova, L. Pereira, V. Fernandes, J. B. Pereira, V. Cerny, Near eastern neolithic genetic input in a small oasis of the Egyptian Western Desert. *Am J Phys Anthropol* **140**, 336-346 (2009).
31. C. Ottoni *et al.*, Comparing maternal genetic variation across two millennia reveals the demographic history of an ancient human population in southwest Turkey. *Royal Society open science* **3**, 150250 (2016).

32. A. J. Drummond, M. A. Suchard, D. Xie, A. Rambaut, Bayesian phylogenetics with BEAUti and the BEAST 1.7. *Molecular Biology and Evolution* **29**, 1969-1973 (2012).
33. W. Clarysse, D. J. Thompson, *Counting the people in Hellenistic Egypt*. Cambridge classical studies (Cambridge University Press, Cambridge ; New York, 2004).
34. I. Lazaridis *et al.*, Ancient human genomes suggest three ancestral populations for present-day Europeans. *Nature* **513**, 409-413 (2014).
35. W. Haak *et al.*, Massive migration from the steppe was a source for Indo-European languages in Europe. *Nature* **522**, 207-211 (2015).
36. I. Lazaridis *et al.*, Genomic insights into the origin of farming in the ancient Near East. *Nature*, (2016).
37. D. H. Alexander, J. Novembre, K. Lange, Fast model-based estimation of ancestry in unrelated individuals. *Genome Res* **19**, 1655-1664 (2009).
38. M. Raghavan *et al.*, Upper Palaeolithic Siberian genome reveals dual ancestry of Native Americans. *Nature* **505**, 87-91 (2014).
39. N. Patterson *et al.*, Ancient admixture in human history. *Genetics* **192**, 1065-1093 (2012).
40. P. R. Loh *et al.*, Inferring admixture histories of human populations using linkage disequilibrium. *Genetics* **193**, 1233-1254 (2013).
41. I. Mathieson *et al.*, Genome-wide patterns of selection in 230 ancient Eurasians. *Nature* **528**, 499-503 (2015).
42. M. R. Falivene, The Herakleopolite Nome: A Catalogue of Toponyms with Introduction and Commentary. *Scholars Press, Atlanta*, (1998).
43. D. W. Rathbone, Villages, land and population in Graeco-Roman Egypt. *Proceecings of the Cambridge Philological Society* **36**, 103-142 (1990).
44. R. Alston, Soldier and Society in Roman Egypt: A Social History. *London/New York, 1995*, (1995).
45. Y. Broux, Depauw, M., Developing Onomastic Gazetteers and Prosopographies for the Ancient World through Named Entity Recognition and Graph Visualization: Some Examples from Trismegistos People. *In Aiello, L.M., McFarland, D. (eds), Social Informatics. Springer*, 304–313 (2015).
46. O. Rubensohn, Griechisch-römische Funde in Ägypten. *Archäologischer Anzeiger* **20**, 65-70 (1905).
47. O. Rubensohn, Knatz, F., Berichte über die Ausgrabungen bei Abusir el Mäläq im Jahre 1903. *Leipzig: Zeitschrift für Ägyptische Sprache und Altertumskunde* **Bd. 41**, 1-21 (1904).
48. R. David, Mummification. In Nicholson, P.T. and Shaw, I. (eds) Ancient Egyptian Materials and Technology. *Cambridge*, 372-389 (2000).
49. G. Lydon, On Trans-Saharan Trails. *Cambridge: Cambridge University Press*, (2009).
50. J. Wright, The Trans-Saharan Slave Trade. *London: Routledge*, (2007).
51. S. T. B. Smith, M.R. , Colonial encounters at New Kingdom Tombos: Cultural entanglements and hybrid identity. In Nubia in the New Kingdom: Lived experience, pharaonic control and local traditions, edited by Neal Spencer, Anna Stevens and Michaela Binder. *British Museum Press, London*, 613-628 (2016).

52. S. T. Smith, Wretched Kush: Ethnic Identities and Boundaries. In Egypt's Nubian Empire. *London: Routledge*, (2003).
53. W. P. Van Pelt, Revising Egypto-Nubian relations in New Kingdom Lower Nubia: From Egyptianization to cultural entanglement. *Cambridge Archaeological Journal* **23**, 523-550 (2013).
54. G. D. Mumford, Egypt and the Levant, The Oxford Handbook of the Archaeology of the Levant: c. 8000-332 BCE. *Oxford (2014)*, (2014).
55. K. I. R. Vandorpe, C. (ed.) The Oxford Handbook of Roman Egypt. *Oxford (2012)*, 260-276 (2012).
56. J. Dabney *et al.*, Complete mitochondrial genome sequence of a Middle Pleistocene cave bear reconstructed from ultrashort DNA fragments. *Proceedings of the National Academy of Sciences of the United States of America* **110**, 15758-15763 (2013).
57. A. Peltzer *et al.*, EAGER: efficient ancient genome reconstruction. *Genome Biol* **17**, 60 (2016).
58. A. McKenna *et al.*, The Genome Analysis Toolkit: a MapReduce framework for analyzing next-generation DNA sequencing data. *Genome research* **20**, 1297-1303 (2010).
59. H. Weissensteiner *et al.*, HaploGrep 2: mitochondrial haplogroup classification in the era of high-throughput sequencing. *Nucleic Acids Res*, (2016).
60. H. Jonsson, A. Ginolhac, M. Schubert, P. L. Johnson, L. Orlando, mapDamage2.0: fast approximate Bayesian estimates of ancient DNA damage parameters. *Bioinformatics* **29**, 1682-1684 (2013).
61. C. Posth *et al.*, Pleistocene Mitochondrial Genomes Suggest a Single Major Dispersal of Non-Africans and a Late Glacial Population Turnover in Europe. *Current biology : CB* **26**, 827-833 (2016).
62. T. S. Korneliussen, A. Albrechtsen, R. Nielsen, ANGSD: Analysis of Next Generation Sequencing Data. *BMC bioinformatics* **15**, 356 (2014).
63. N. Patterson, A. L. Price, D. Reich, Population structure and eigenanalysis. *PLoS genetics* **2**, e190 (2006).
64. K. Tamura, M. Nei, Estimation of the number of nucleotide substitutions in the control region of mitochondrial DNA in humans and chimpanzees. *Mol Biol Evol* **10**, 512-526 (1993).
65. D. Darriba, G. L. Taboada, R. Doallo, D. Posada, jModelTest 2: more models, new heuristics and parallel computing. *Nat Methods* **9**, 772 (2012).
66. Y. Benjamini, Y. Hochberg, Controlling the false discovery rate: a practical and powerful approach to multiple testing. *Journal of the Royal Statistical Society. Series B (Methodological)*, 289-300 (1995).
67. M. Slatkin, A measure of population subdivision based on microsatellite allele frequencies. *Genetics* **139**, 457-462 (1995).
68. P. B. Frandsen, B. Calcott, C. Mayer, R. Lanfear, Automatic selection of partitioning schemes for phylogenetic analyses using iterative k-means clustering of site rates. *BMC evolutionary biology* **15**, 13 (2015).
69. F. Jia, N. Lo, S. Y. Ho, The impact of modelling rate heterogeneity among sites on phylogenetic estimates of intraspecific evolutionary rates and timescales. *PloS one* **9**, e95722 (2014).

70. A. S. Rambaut, M.A.; Xie, D.; Drummond, A.J., Tracer v1.6, Available from http://beast.bio.ed.ac.uk/Tracer. (2014).
71. D. Reich *et al.*, Reconstructing Native American population history. *Nature* **488**, 370-374 (2012).
72. J. N. Fenner, Cross-cultural estimation of the human generation interval for use in genetics-based population divergence studies. *Am J Phys Anthropol* **128**, 415-423 (2005).
73. H. Li, R. Durbin, Fast and accurate short read alignment with Burrows-Wheeler transform. *Bioinformatics* **25**, 1754-1760 (2009).
74. D. M. Behar *et al.*, A "Copernican" reassessment of the human mitochondrial DNA tree from its root. *Am J Hum Genet* **90**, 675-684 (2012).
75. M. van Oven, M. Kayser, Updated comprehensive phylogenetic tree of global human mitochondrial DNA variation. *Hum Mutat* **30**, E386-394 (2009).
76. H. Wickham, *ggplot2: Elegant Graphics for Data Analysis*. Springer (2009).
77. M. F. Hammer *et al.*, The geographic distribution of human Y chromosome variation. *Genetics* **145**, 787-805 (1997).
78. M. F. Hammer *et al.*, Hierarchical patterns of global human Y-chromosome diversity. *Mol Biol Evol* **18**, 1189-1203 (2001).
79. M. F. Hammer *et al.*, Out of Africa and back again: nested cladistic analysis of human Y chromosome variation. *Mol Biol Evol* **15**, 427-441 (1998).
80. F. Cruciani *et al.*, A back migration from Asia to sub-Saharan Africa is supported by high-resolution analysis of human Y-chromosome haplotypes. *Am J Hum Genet* **70**, 1197-1214 (2002).
81. T. Bersaglieri *et al.*, Genetic signatures of strong recent positive selection at the lactase gene. *Am J Hum Genet* **74**, 1111-1120 (2004).
82. N. S. Enattah *et al.*, Identification of a variant associated with adult-type hypolactasia. *Nature genetics* **30**, 233-237 (2002).
83. M. Soejima, Y. Koda, Population differences of two coding SNPs in pigmentation-related genes SLC24A5 and SLC45A2. *International journal of legal medicine* **121**, 36-39 (2007).
84. R. Kimura *et al.*, A common variation in EDAR is a genetic determinant of shovel-shaped incisors. *Am J Hum Genet* **85**, 528-535 (2009).
85. A. Fujimoto *et al.*, A scan for genetic determinants of human hair morphology: EDAR is associated with Asian hair thickness. *Human molecular genetics* **17**, 835-843 (2008).
86. H. Eiberg *et al.*, Blue eye color in humans may be caused by a perfectly associated founder mutation in a regulatory element located within the HERC2 gene inhibiting OCA2 expression. *Human genetics* **123**, 177-187 (2008).
87. R. A. Sturm *et al.*, A single SNP in an evolutionary conserved region within intron 86 of the HERC2 gene determines human blue-brown eye color. *Am J Hum Genet* **82**, 424-431 (2008).
